# Longitudinal trajectories of disability among Chinese adults: the role of cardiometabolic multimorbidity

**DOI:** 10.1007/s40520-024-02732-8

**Published:** 2024-03-23

**Authors:** Huihui He, Raoping Tu, Huahua Chen, Chao Wang, Shengjuan Wu, Suhang Wang

**Affiliations:** 1 Department of Nursing, The First Affiliated Hospital of Wannan Medical College, Wuhu, Anhui China; 2 School of Health Management, Fujian Medical University, Fuzhou, Fujian China; 3 Department of Gastrointestinal surgery, The First Affiliated Hospital of Wannan Medical College, Wuhu, Anhui China; 4 Anesthesia Surgery and Pain Management, Department Zhongda Hospital, School of Medicine Southeast University, Nanjing, Jiangsu China

**Keywords:** Cardiometabolic multimorbidity, Disability, Trajectory, Diabates, Stroke, Heart diseases

## Abstract

**Background:**

Cardiometabolic multimorbidity (CM) has been found to be associated with higher mortality and functional limitations. However, few studies have investigated the longitudinal association between CM and disability in the Chinese population and whether these associations vary by smoking status.

**Methods:**

The study included 16,754 participants from four waves (2011, 2013, 2015, and 2018) of China Health and Retirement Longitudinal Study (CHARLS) (mean age: 59, female: 51%). CM was assesed at baseline and defined as having two or more of diabetes, stroke, or heart disease. Disability was repeatedly measured by summing the number of impaired activities of daily living (ADL) and instrumental activities of daily living (IADL) during the 7-year follow-up. Linear mixed-effects model was used to determine the association of CM and trajectories of disability and to assess the modification effect of smoking status in these associations.

**Results:**

Participants with CM at baseline had a faster progression of disability compared to those without CM (CM: β = 0.13, 95% CI: 0.05 to 0.21). Current smokers with CM developed disability faster than their counterparts (P_interaction for smoking_=0.011). In addition, there was a significant association between CM and the annual change of disability in current smokers (β = 0.34, 95% CI: 0.17 to 0.50) while no such association was observed in current non-smokers (β = 0.08, 95% CI: -0.02 to 0.17).

**Conclusion:**

CM was associated with more a rapid disability progression. Notably, being current smokers may amplify the adverse effects of CM on disability progression.

**Supplementary Information:**

The online version contains supplementary material available at 10.1007/s40520-024-02732-8.

## Introduction

Disability, defined as difficulty in performing activities necessary for independent living, has been increasing, a global trend partly driven by increasingly aging population and diseases [1]. Available studies suggest its association with adverse health outcomes, including increased health care utilization, low quality of life, and high risk of mortality [2–4]. Chronic non-communicable disease has attracted increasing attentions, with studies reporting a correlation between chronic disease and a higher risk of disability, especially in middle-aged and older populations, thus necessitating modulation to prevent or delay the progression of disability [5].

Non-communicable diseases, especially cardio-metabolic diseases, are rapidly increasing in low- and middle-income countries [6]. Cardiometabolic multimorbidity (CM) is the simultaneous presence of two or more cardiometabolic diseases (diabetes, stroke, and heart disease), which is rapidly increasing as the population ages [7]. The Emerging Risk Factors Collaboration (ERFC) reported that any combination of cardiometabolic diseases was associated with multiplicative mortality risk, with each additional disease doubling the risk of mortality [7]. Additionally, exposure to CM was shown to increase the odds of activity limitation among Canada adults [8]. In the Chinese population, however, no relative correlation between the above-mentioned CM and disability have been explored.

Smoking is one of the major threats to global public health as well as a leading risk factor for premature death and disability from non-communicable diseases in Chinese [9, 10]. Additionally, previous research has supported that smoking increases the risk of cardiovascular disease, stroke, and diabetes [11, 12]. This poor lifestyle with respect to tobacco exposure may play a role in the association between CM and activity limitation.

In view of these considerations, we aimed to explore the effect of CM on the progression of disability and to explore the possible role of smoking status through a nationally representative cohort-China Health and Retirement Longitudinal Study (CHARLS).

## Method

**Fig. 1:**
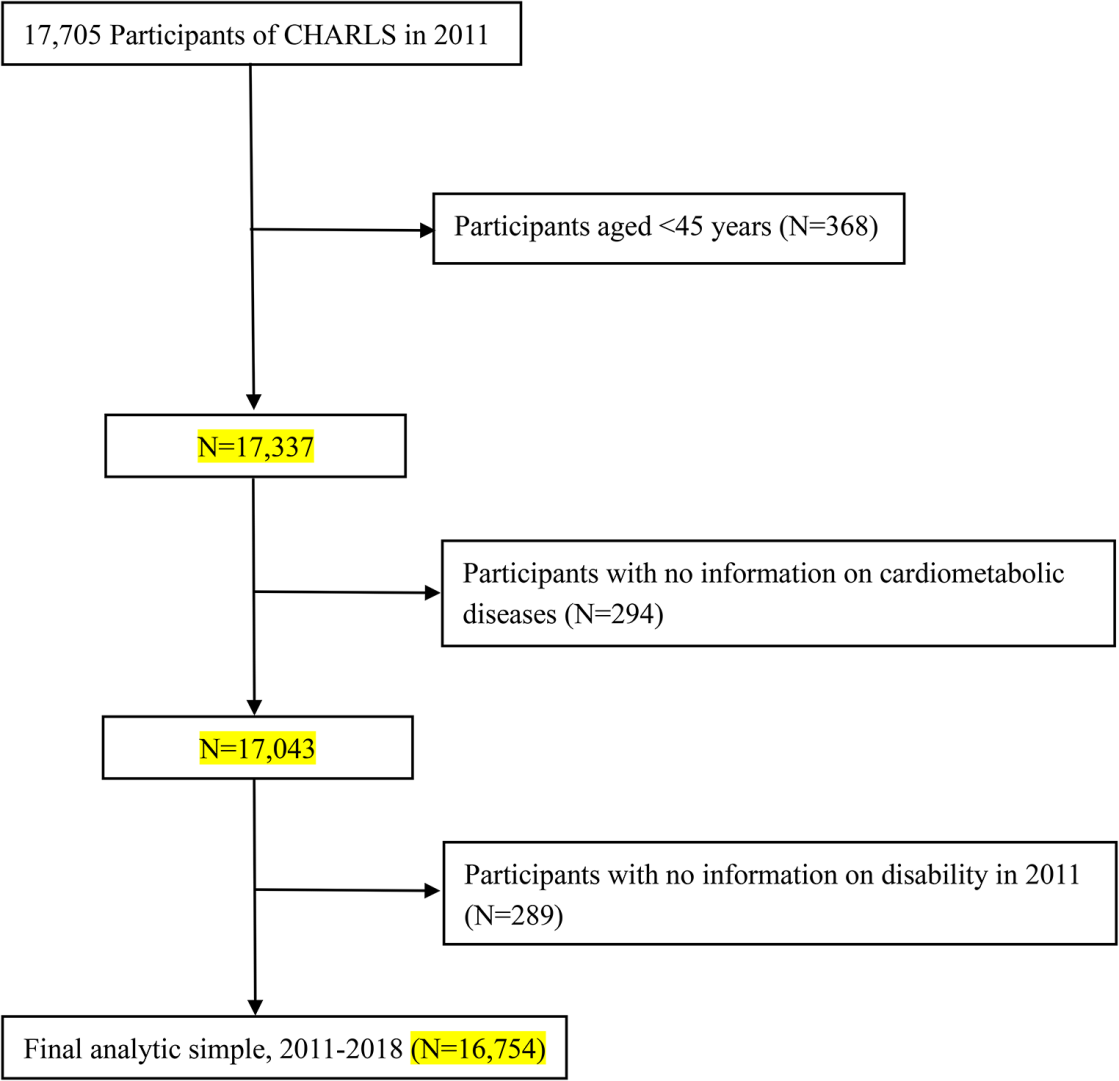
Flowchart of study participants

Data were obtained from the China Health and Retirement Longitudinal Study (CHARLS) that is a nationally representative cohort survey based on community residents aged 45 years and older. Using a multi-stage probability sampling method, the baseline survey began in 2011 with a follow-up survey conducted every two years, including a biennial face-to-face interview to assess the social, economic and health status of participants and blood sample collected once in every two follow-up periods [13]. In this study, we used four waves (2011, 2013, 2015, and 2018) of CHARLS. Those who (1) younger than 45 years old (*n* = 368); (2) with no information on diabetes, stroke and heart diseases (*n* = 294); (3) with no information on disability at baseline (*n* = 289) were excluded, leaving 16,754 participants to be remained in the analytical sample (Fig. 1).

### Measurements

#### Cardiometabolic multimorbidity

CM was assessed at baseline and defined as having two or more of diabetes, stroke, or heart disease [7]. Diabetes was identified when having one of the following conditions: (1) fasting plasma glucose (FPG) ≥ 126 mg/dl; (2) glycated hemoglobin (HbA1c) ≥ 6.5%; (3) self-reported doctor diagnosed diabetes; (4) diabetes treatment [14]. Fasting plasma glucose concentration was measured by enzymatic colorimetric analysis and glycated hemoglobin concentration was determined by borate affinity high performance liquid chromatography [15]. Stroke or heart disease (e.g., heart attack, coronary heart disease, angina, congestive heart failure, or other heart problems) were identified if participants reported the specific disease diagnoses or treatment.

#### Disability

Activities of daily living (ADL) (6 items: eating, bathing, dressing, getting in and out of bed, toileting, and controlling urination and defecation) and instrumental activities of daily living (IADL) (5 items: doing household chores, preparing meals, shopping, managing money, and taking medications) were assessed using participant self-reported information at baseline and every follow-up visits [16, 17]. Each item has four options, participants reporting no difficulty are considered 0 point, otherwise 1 point. We first considered ADL and IADL as separate scales and then merged them together (range:0–11) for enhancing range and sensitivity of the measurement [18].

#### Covariates

Sociodemographic (age, sex, education, marital, residential location), behavioral habits (smoking and alcohol consumption) and health information (other non-communicable diseases, depressive symptoms, body mass index) were considered as covariates and measured at baseline. All these factors were considered associated with disability and were regarded as control covariates [19, 20].

Education was classified as ‘≤ 6 years’ and ‘> 6 years’ based on the maximum years of schooling, marital status was categorized into married and non-married (separated/ divorced/widowed/single). Residential location was assessed according to living area: ‘rural’ versus ‘urban’, smoking was dichotomized as current non-smokers (including former smokers) and current smokers. Alcohol consumption was classified as occasional drinkers (less than or equal to three times a week) and habitual drinkers (more than three times a week). Based on participants’ self-reported doctor diagnoses of certain diseases or treatments for these diseases, other non-communicable diseases (NCDs) was divided into two groups: ‘yes’ and ‘no’. Depression symptoms were measured using the 10-item Center for Epidemiologic Studies Depression Scale (ranging 0 to 30) [21]. Body mass index (BMI) was calculated by dividing weight (kg) by height squared (m^2^) and defined as a continuous variable.

### Statistical analysis

First, student ‘s t - test for numerical variables and chi-square tests for categorical variables were used to compare sociodemographic and health-related characteristics of participants with and without CM. Second, linear mixed-effects model was used to estimate the association between CM and the trajectory of disability over time. CM status, follow-up time, and their interactions were considered as fixed effects, while individual differences in baseline disability and in the rate of disability accumulation were regarded as random effects. The interaction between time and CM status represents the estimated difference in the annual rate of disability progression between CM group and non-CM group. Covariates including age, sex, education, marital, residential location, smoking, alcohol consumption, NCDs, depressive symptoms, and BMI were adjusted in the analysis.

Third, further analysis with a time-by-CM-by-smoking interaction was included to test the modification effect of smoking in the longitudinal association between CM and disability.

In addition, a number of sensitive analyses were conducted by repeating the analyses: (1) adjusting cognitive function as a confounding factor; (2) excluding those who had memory-related disease at baseline (*n* = 122) to reduce recall bias; (3) excluding those who had ADL or IADL disability at baseline (*n* = 2751) to minimize the influence of reverse causation.

A two-tailed *P* < 0.05 was considered statistically significant. All analysis were performed using STATA 15.0.

## Results

Table 1 showed the baseline characteristics of participants classified by CM status. A total of 16,754 participants were included in our study, with a mean age of 59 years old and 51% were female. Among them, 560 (3.3%) reported having CM at baseline. Compared to participants without CM, those with CM were more likely to be females, older, current non-smokers, occasional drinkers, likely to have higher BMI, NCDs, and experience more disabilities and depressive symptoms. However, we did not observe significant differences in education level, marital status, and place of residence between participants with and without CM. In addition, participants with missing values on CM or/and disabilities as well as those who dropped out tended to be older, males, occasional drinkers, have more depressive symptoms and disabilities, have higher education levels, lower BMI, and live in urban areas, and less likely to be married compared to those with complete information (Table S1).

**Table 1:** Baseline characteristics of participants aged 45 years and older by CM status in 2011

	Total (*n* = 16,754)	non-CM (*n* = 16,194)	CM (*n* = 560)	P
Age (years), mean (*SD*)	59.41 ± 9.88	59.27 ± 9.86	63.41 ± 9.49	< 0.001
Sex, n (%)				0.003
Female	8577 (51.23)	8256 (51.02)	321 (57.42)	
Male	8165 (48.77)	7927 (48.98)	238 (42.58)	
Education				0.506
≤ 6 years	14,584 (87.15)	14,102 (87.18)	482 (86.23)	
> 6 years	2150 (12.85)	2073 (12.82)	77 (13.77)	
Marital status				0.286
Married	14,604 (87.17)	14,125 (87.22)	479 (85.69)	
Non-married	2149 (12.83)	2069 (12.78)	80 (14.31)	
Residential location				< 0.001
Rural	12,409 (75.30)	12,104 (75.93)	305 (56.69)	
Urban	4070 (24.70)	3837 (24.07)	233 (43.31)	
Smoking				< 0.001
Current non-smokers	11,398 (70.62)	10,958 (70.26)	440 (80.88)	
Current smokers	4743 (29.38)	4639 (29.74)	104 (19.12)	
Alcohol consumption				< 0.001
Occasional drinkers	13,735 (88.13)	13,224 (87.89)	511 (94.81)	
Habitual drinkers	1850 (11.87)	1822 (12.11)	28 (5.19)	
BMI (kg/m^2^), mean (*SD*)	23.39 ± 3.65	23.31 ± 3.61	25.53 ± 3.93	< 0.001
NCDs				< 0.001
Yes	10,915 (65.62)	10,397 (64.67)	518 (92.83)	
No	5719 (34.38)	5679 (35.33)	40 (7.17)	
Depressive symptoms				< 0.001
No depressive symptoms	9238 (62.96)	9014 (63.55)	224 (45.90)	
Depressive symptoms	5434 (37.04)	5170 (36.45)	264 (54.10)	
Total (ADL + IADL) disabilities	0.84 ± 1.97	0.80 ± 1.90	2.2 ± 3.17	< 0.001

*Notes* Data are presented as mean (standard deviations) or number (proportion %). CM, cardiometabolic multimorbidity; BMI, body mass index; NCDs, non-communicable diseases; ADL, activities of daily living; IADL, instrumental activities of daily living. Missing values: 5 missing in age, 12 missing in sex, 20 missing in education, 275 missing in residential location, 613 missing in smoking, 1169 missing in alcohol consumption, 3755 missing in BMI, 524 missing in NCDs, 2082 missing in depressive symptoms

**Fig. 2:**
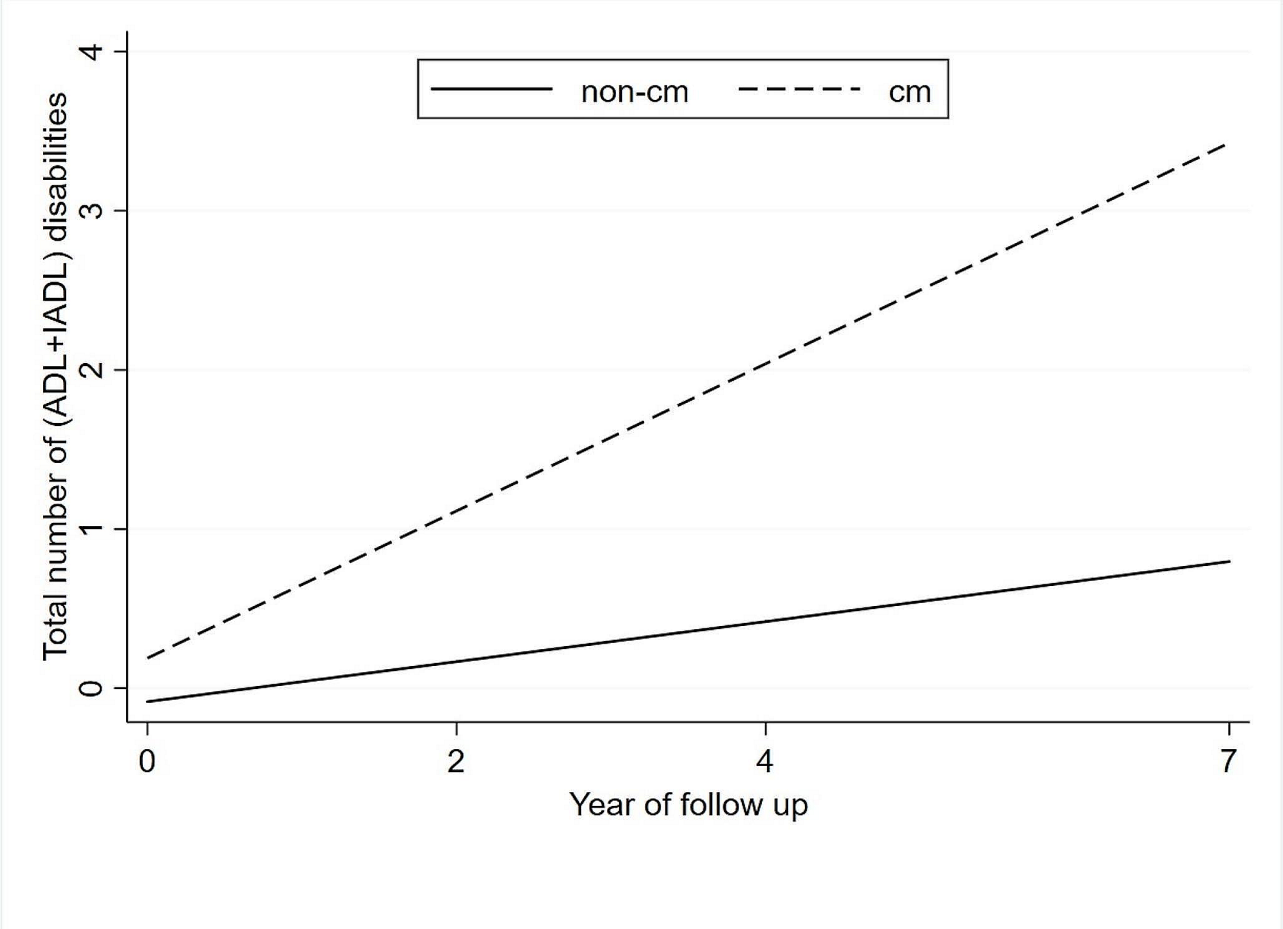
Trajectories of total (ADL + IADL) disabilities over 7 years by cardiometabolic multimorbidity status

The results from linear mixed-effects model that estimated associations between CM status and ADL and IADL disability trajectories were presented in Table 2. The average (ADL + IADL) disability scores increased over time from 0.8 in 2011, 0.8 in 2013, 1.0 in 2015 to 1.1 in 2018 (data not shown). Participants with CM had higher baseline disability scores compared to those without CM (β = 0.57, 95%CI: 0.41 to 0.73). In the fully adjusted model, compared to people with non-CM, those who with CM had faster disability progression (β = 0.13, 95%CI: 0.05 to 0.21) within seven years (Fig. 2). In addition, similar trend was observed when ADL and IADL were regarded as dependent variables, respectively (β = 0.07, 95%CI: 0.03 to 0.12; β = 0.05, 95%CI: 0.01 to 0.10) (Table 2).

**Table 2:** Association between baseline cardiometabolic multimorbidity and changes in the number of ADL and IADL disabilities over 7 years

CM status	(ADL + IADL)		ADL		IADL	
	β (95%CI)	P	β (95%CI)	P	β (95%CI)	P
Time, years	0.13 (0.12 to 0.15)	< 0.001	0.07 (0.06 to 0.08)	< 0.001	0.07 (0.06 to 0.08)	< 0.001
Intercept						
non-CM	Reference		Reference		Reference	
CM	0.57 (0.41 to 0.73)	< 0.001	0.27 (0.18 to 0.36)	< 0.001	0.30 (0.20 to 0.39)	< 0.001
Slope						
Time × non-CM	Reference		Reference		Reference	
Time × CM	0.13 (0.05 to 0.21)	< 0.001	0.07 (0.03 to 0.12)	0.001	0.05 (0.01 to 0.10)	0.018

*Notes* Adjusted for age, sex, education, marital, residential location, smoking, alcohol consumption, body mass index, other non-communicable diseases and depressive symptoms. CM, cardiometabolic multimorbidity; CI, confidence interval; ADL, activities of daily living; IADL, instrumental activities of daily living

Table 3 showed the moderating role of smoking status in the relationship between CM and disability trajectory. There was a significant interaction between smoking and CM on the annual-year change of disability (P _interaction for smoking_=0.011). That is, baseline CM was significantly associated with changes in the number of ADL and IADL disabilities in current smokers but not in current non-smokers. Table 3 showed that for current non-smokers with and without CM, the rate of increase in the number of disabilities over time was comparable, while current smokers with CM had a higher rate of increase in disability over time than current smokers without CM.

**Table 3:** Association between baseline cardiometabolic multimorbidity and changes in the number of ADL and IADL disabilities over 7 years by smoking status

	β (95%CI)	P
Current non-smokers		
Time × non-CM	Reference	
Time × CM	0.08 (-0.02 to 0.17)	0.102
Current smokers		
Time × non-CM	Reference	
Time × CM	0.34 (0.17 to 0.50)	< 0.001
CM×Smoking×Time	0.26 (0.06 to 0.46)	0.011

*Notes* Model was adjusted for age, sex, education, marital, residential location, alcohol consumption, body mass index, other non-communicable diseases, and depressive symptoms. CM, cardiometabolic multimorbidity; CI, confidence interval; ADL, activities of daily living; IADL, instrumental activities of daily living

### Sensitivity analysis

Similar tendency was found in the analysis when adjusting cognitive function (Table S2). Moreover, the associations between CM and trajectories of disability were similar among those without memory-related disease or disability at baseline (Table S3 and S4).

## Discussion

In this community-based cohort study, we found that the presence of CM was associated with a more rapid increasing rate in the total number of (ADL + IADL) disabilities during the 7-year follow-up period among Chinese middle-aged and older adults after adjusting for potential confounding factors. In addition, there was a multiplicative interaction between CM and smoking status on disability progression, indicating that being current smokers may exacerbate the deleterious effect of CM on disability progression in middle-aged and older adults. Our current findings in a general population might be helpful in improving the health-care plan for individuals with CM, aiming to slow down the progression of disability in middle-aged and older adults.

Our findings are consistent with Brayden’s study that they have examined the cross-sectional association between CM and (ADL + IADL) limitations by using data from the Canadian Longitudinal Study on Aging [8], and we extend their results by demonstrating the predictive role of CM in the longitudinal trajectory of disability. Furthermore, a previous report by the The China Health and Nutrition Survey demonstrated the longitude correlation between CM and higher risk of ADL limitations [22]. Additionally, in our study, individuals with CM were more likely to be current non-smokers or occasional drinkers, possibly because a portion of individuals with CM were former smokers (17.63%) (have quit smoking) or former drinkers (20.17%). Due to illness, they had already quit some unhealthy habits (e.g., smoking or drinking) at baseline.

The underlying mechanisms linking cardiac metabolic diseases to ADL/IADL disability accumulation can be explained in the following aspects. For one thing, a high-risk state for disability development is based on diminished homeostatic capacity across multiple physiological systems [23]. For example, high glucose levels in diabetes patients can damage cardiac blood vessels, promote the aggregation of arterial plaques, inflammatory responses, and oxidative stress [24]. These factors can lead to the development of atherosclerosis and an increased risk of thrombosis, that subsequently leads muscle catabolism and disability as part of the frailty process [25, 26]. For another, impairment of heart function may result in exacerbated stroke consequences, including increased stroke size and heightened neurological deficits, thus accelerating the accumulation of disability [27].

Furthermore, the present study showed that association between the CM and disability accumulation was modified by smoking status, with current smokers experiencing the highest risk. Possible explanations might be that the interaction between CM and smoking may lead to more severe inflammation and oxidative stress, further impairing bodily functions, resulting in the accumulation of limitations in ADL/IADL [28–30]. Our results provide further evidence that special attention should be directed towards current smokers among individuals with CM, as they are important targets for reducing the accumulation of disabilities.

### Strengths and limitations

The strengths of this study lie in its utilization of a large sample of population-based data and its prospective design, as well as the repeated measurements of outcomes (i.e., the counting of ADL and IADL limitations), that make the study with increased precision and generalizability. Moreover, the combination of ADL and IADL could help screen a wider range of disability and enhance the sensitivity of the measurement [18]. However, there are several limitations to this study. First, CM was measured only at baseline, therefore, we were unable to assess disability progression related to changes in the CM over time. Disability may have accumulated faster in individuals without CM at baseline due to new onset of CM. Biases would not have occurred if the number of CM increased at a comparable rate over time. However, due to ceiling effects in those with CM at baseline, we may have underestimated the associations of the CM with disability progression by not updating the CM status over time. Future research is needed to monitor the trajectories of the CM and examine their added prognostic value. Second, information regarding heart disease, stroke, ADL, IADL, and some covariates were self-reported, which may introduce reporting bias and potentially impact the risk estimates in this study and therefore our results need to be interpreted with caution. Third, missing values on CM could potentially introduce selection bias. Nevertheless, the missing values of CM were less (1.8%), which may not affect the overall results. Finally, longitudinal data are often incomplete or unbalanced due to loss of follow-up. However, linear mixed-effect models are capable of handling missing data and inconsistent measurement intervals within and across participants, allowing us to utilize all available data for each participant [31].

## Conclusion

In this prospective study, CM was associated with disability accumulation, and this association was moderated by smoking status, with current smokers experiencing a more rapid progression of disabilities. Therefore, it is important to implement disability-prevention measures aimed at reducing smoking among middle-aged and older individuals with cardiometabolic multimorbidity.

### Electronic supplementary material

Below is the link to the electronic supplementary material.

Supplementary Material 1

## Data Availability

The dataset collected in the current study are available from the website: http://charls.pku.edu.cn/index/en.html.
