# Supplementary material for: Longitudinal trajectories of disability among Chinese adults: the role of cardiometabolic multimorbidity
Source: Aging Clin Exp Res. 2024 Mar 23;36(1):79. doi: 10.1007/s40520-024-02732-8 (PMC10960913; doi:10.1007/s40520-024-02732-8)
Supplement: Supplementary file 1 — Supplementary Material 1 [file 40520_2024_2732_MOESM1_ESM.docx]

| **Table S1** Baseline characteristics of participants with and without missing values on CM or/and disabilities and those who dropped out or not | | | |
| --- | --- | --- | --- |
|  | Missing (n=6,458) | Non-missing (n=10,879) | P |
| Age (years), mean (*SD*) | 61.14±11.35 | 58.41±8.81 | <0.001 |
| Sex, n (%) |  |  | <0.001 |
| Female | 3167 (49.08) | 5726 (52.67) |  |
| Male | 3286 (50.92) | 5146 (47.33) |  |
| Education |  |  | <0.001 |
| ≤6 years | 5442 (84.87) | 9631 (88.58) |  |
| >6 years | 970 (15.13) | 1242 (11.42) |  |
| Marital status |  |  | <0.001 |
| Married | 5297 (82.41) | 9752 (89.64) |  |
| Non-married | 1131 (17.59) | 1127 (10.36) |  |
| Residential location |  |  | <0.001 |
| Rural | 4119 (65.10) | 8712 (81.21) |  |
| Urban | 2208 (34.90) | 2016 (18.79) |  |
| Smoking |  |  | 0.075 |
| Current non-smokers | 4260 (71.57) | 3149 (29.74) |  |
| Current smokers | 1692 (28.43) | 7439 (70.26) |  |
| Alcohol consumption |  |  | 0.007 |
| Occasional drinkers | 5165 (89.11) | 8922 (87.67) |  |
| Habitual drinkers | 631 (10.89) | 1255 (12.33) |  |
| BMI (kg/m^2^), mean (*SD*) | 23.09±3.66 | 23.51±3.63 | <0.001 |
| NCDs |  |  | 0.243 |
| Yes | 4144 (66.27) | 7061 (65.39) |  |
| No | 2109 (33.73) | 3737 (34.61) |  |
| Depressive symptoms |  |  | 0.040 |
| No depressive symptoms | 3192 (61.84) | 6222 (63.54) |  |
| Depressive symptoms | 1970 (38.16) | 3570 (36.46) |  |
| Total (ADL+IADL) disabilities | 1.18±2.45 | 0.67±1.62 | <0.001 |

*Notes:* Data are presented as mean (standard deviations) or number (proportion %). CM, cardiometabolic multimorbidity; BMI, body mass index; NCDs, non-communicable diseases; ADL, activities of daily living; IADL, instrumental activities of daily living.

*Notes:* Adjusted for age, sex, education, marital, residential location, smoking, alcohol consumption, body mass index, non-communicable diseases, depressive symptoms, and cognitive function. CM, cardiometabolic multimorbidity; CI, confidence interval; ADL, activities of daily living; IADL, instrumental activities of daily living.

| **Table S2** Association between baseline cardiometabolic multimorbidity and changes in the number of ADL and IADL disabilities over 7 years after adjusting cognitive function | | | | | | |
| --- | --- | --- | --- | --- | --- | --- |
| **CM status** | **(ADL+IADL)** |  | **ADL** |  | **IADL** |  |
|  | β (95%CI) | P | β (95%CI) | P | β (95%CI) | P |
| Time, years | 0.12 (0.11 to 0.14) | <0.001 | 0.06 (0.05 to 0.07) | <0.001 | 0.07 (0.06 to 0.07) | <0.001 |
| **Intercept** |  |  |  |  |  |  |
| non-CM | Reference |  | Reference |  | Reference |  |
| CM | 0.42 (0.26 to 0.59) | <0.001 | 0.19 (0.10 to 0.28) | <0.001 | 0.23 (0.13 to 0.33) | <0.001 |
| **Slope** |  |  |  |  |  |  |
| Time × non-CM | Reference |  | Reference |  | Reference |  |
| Time × CM | 0.16 (0.07 to 0.25) | <0.001 | 0.08 (0.04 to 0.13) | 0.001 | 0.07 (0.02 to 0.12) | 0.004 |

| **Table S3** Trajectories of total (ADL + IADL) disabilities over 7 years in relation to cardiometabolic multimorbidity among those without memory-related disease at baseline | | | | | | |
| --- | --- | --- | --- | --- | --- | --- |
| **CM status** | **(ADL+IADL)** |  | **ADL** |  | **IADL** |  |
|  | β (95%CI) | P | β (95%CI) | P | β (95%CI) | P |
| Time, years | 0.14 (0.12 to 0.15) | <0.001 | 0.07 (0.06 to 0.08) | <0.001 | 0.07 (0.06 to 0.08) | <0.001 |
| **Intercept** |  |  |  |  |  |  |
| non-CM | Reference |  | Reference |  | Reference |  |
| CM | 0.51 (0.35 to 0.68) | <0.001 | 0.24 (0.15 to 0.33) | <0.001 | 0.27 (0.17 to 0.36) | <0.001 |
| **Slope** |  |  |  |  |  |  |
| Time × non-CM | Reference |  | Reference |  | Reference |  |
| Time × CM | 0.13 (0.05 to 0.22) | 0.001 | 0.07 (0.02 to 0.12) | 0.002 | 0.06 (0.02 to 0.11) | 0.009 |

*Notes:* Adjusted for age, sex, education, marital, residential location, smoking, alcohol consumption, body mass index, non-communicable diseases, and depressive symptoms. CM, cardiometabolic multimorbidity; CI, confidence interval; ADL, activities of daily living; IADL, instrumental activities of daily living.

| **Table S4** Trajectories of total (ADL + IADL) disabilities over 7 years in relation to cardiometabolic multimorbidity among those without disability at baseline | | | | | | |
| --- | --- | --- | --- | --- | --- | --- |
| **CM status** | **(ADL+IADL)** |  | **ADL** |  | **IADL** |  |
|  | β (95%CI) | P | β (95%CI) | P | β (95%CI) | P |
| Time, years | 0.25 (0.24 to 0.27) | <0.001 | 0.11 (0.10 to 0.12) | <0.001 | 0.14 (0.13 to 0.15) | <0.001 |
| **Intercept** |  |  |  |  |  |  |
| non-CM | Reference |  | Reference |  | Reference |  |
| CM | -0.04 (-0.16 to 0.07) | 0.462 | -0.03 (-0.10 to 0.04) | 0.370 | -0.02 (-0.09 to 0.05) | 0.573 |
| **Slope** |  |  |  |  |  |  |
| Time × non-CM | Reference |  | Reference |  | Reference |  |
| Time × CM | 0.31 (0.22 to 0.40) | <0.001 | 0.14 (0.09 to 0.19) | <0.001 | 0.17 (0.12 to 0.22) | <0.001 |

*Notes:* Adjusted for age, sex, education, marital, residential location, smoking, alcohol consumption, body mass index, non-communicable diseases, and depressive symptoms. CM, cardiometabolic multimorbidity; CI, confidence interval; ADL, activities of daily living; IADL, instrumental activities of daily living.
